# Bilateral Bone Ridge Splitting in Maxilla with Immediate Implant Placement in a Patient with Osteoporosis: A Clinical Report with 2-Year Follow-up

**DOI:** 10.1155/2019/1458571

**Published:** 2019-06-16

**Authors:** Rafał Flieger

**Affiliations:** Private Dental Healthcare, ul. Nacławska 11, 64-000 Kościan, Poland

## Abstract

Every year, a higher percentage of bisphosphonates is prescribed for osteoporosis treatment which can lead to bone osteonecrosis after several surgical procedures in the oral cavity. This report describes an approach to restore two missing teeth, employing bilateral bone ridge splitting in the maxilla with immediate placement of implants in a patient using bisphosphonates in the management of osteoporosis. Two titanium implants with a width of 3.45 mm and a length of 10 mm were placed in the maxillary ridge with a diameter of 4.4 mm and 3.0 mm in positions 15 and 24 according to the classification of the World Dental Federation. The implants were placed immediately by bone splitting, using a piezosurgery device and guided bone regeneration with an alloplastic material and a collagen membrane. Five months later, the implants were uncovered and the final porcelain crowns were cemented. 24 months later, the control through clinical and radiographical examinations showed no bone loss in the collar part of the implants and the proper status of the peri-implant soft tissue without any signs of inflammation. Piezosurgery is a useful and safe method of ridge splitting in a very thin ridge (4.5 and 3.0 mm).

## 1. Introduction

The rapid development of implant dentistry has resulted in improvements in the characteristics and design of titanium implants achieving a high level of bone-to-implant contact; therefore, the osseointegration process no longer poses a challenge [1]. The recent literature confirmed that 99.1% of implants in the mandible and 84.9% of implants in the maxilla have become osseointegrated and have allowed for the prosthetic reconstruction of missing teeth in a 5-year follow-up [2]. The complication rate of this treatment varies from 0% to 29% after 5- and 10-year follow-ups, respectively [3, 4]. The essential factor conditioning the implant insertion success is the availability of sufficient amounts of soft and hard tissues [5–7]. In cases of a lack of proper bone thickness of the alveolar ridge, an additional surgical procedure is required. Augmentation of thin alveolar ridges can be accomplished by several techniques, such as by bone block grafting and by ridge splitting [8].

The method of ridge splitting was introduced for the first time by Tatum in 1969 [9]. The study of Sethi and Kaus showed implant survival rates of 97-98.8% after five years [10]. Bone splitting using different devices, such as the piezo saw and osteotome, allows for implant insertion even with a ridge thinner than 3.5 mm with only a minimal risk of a bone plate fracture or perforation. The lateral ridge split augmentation technique, with immediate implant placement, reduces the time of the treatment and the time of the final prosthetic reconstruction. Furthermore, compression of the bone increases its density [11]. Lateral crest splitting can be performed using various surgical tools, e.g., chisels, rotary burs, and saws, or using the piezosurgery unit [8]. The piezosurgery machine produces ultrasounds with a frequency in the range of 22–35 kHz and affords surgical precision and safeness of cutting with minimal bleeding [8]. The relative disadvantage of this technique is the increase in bone temperature during osteotomy; hence, adequate cooling and the high-level skills of the operator are demanded [12, 13].

The success of the implant treatment is affected by the quality and quantity of the soft and hard tissues and the characteristics of the titanium implants [1, 5–8, 14]. However, patient-related factors are also crucial, especially their health status. Every year, a higher percentage of antiresorptive and antiangiogenetic medicines are prescribed for osteoporosis treatment. Specific remedies hinder osteoclastic action, thereby reducing bone turnover, which raises the patients' quality of life [15]. The administration of antiresorptive and antiangiogenetic medicines, especially in oncology, diminishes the incidence of bone fracture for patients with osteoporosis [16]. However, a serious side effect that can develop as a result of this therapy is medication-related osteonecrosis of the jaws (MRONJ) and in particular bisphosphonate-related osteonecrosis of the jaw (BRONJ), the first form of this side effect, described by Marx in 2003 [17].

In 2014, the American Association of Oral and Maxillofacial Surgeons define MRONJ as jawbone osteonecrosis related to antiresorptive and antiangiogenetic drugs, describing its main features as follows: bone exposure in the maxillofacial region that persisted for more than 8 weeks, patient treated with antiresorptive or antiangiogenic medications, no history of radiation, and no traumatic origin of bone exposure [15].

This article describes an approach to restore two missing teeth employing bilateral bone ridge splitting in the maxilla with immediate implant placements in a patient using bisphosphonates in the management of osteoporosis.

## 2. Case Presentation

### 2.1. Presurgical Phase

A 56-year old white woman was referred to our clinic for prosthetic crown reconstruction of two missing molars in the maxilla. The patient lost her teeth (15, 24 FDI) 15 months ago because of a deep carious lesion. The patient was not using any dental prostheses post the extractions. Medical history revealed that she suffered from hypertension and osteoporosis, which were all under medical control. For her osteoporosis, the patient used oral bisphosphonate of alendronate (Fosamax) at a dose of 70 mg/week for 24 months. During the time of osteoporosis treatment with alendronate, there were no accidents of bone osteonecrosis following the teeth's extraction.

After a consultation with the medical doctor of the patient and reviewing the literature concerning surgical therapy in patients with osteoporosis, we decided to perform the placement of the implants without stopping the bisphosphonate therapy. However, before the operation the patient was given antibiotic treatment with amoxicillin+clavulanic acid (Amoxiclav, Sandoz, Poland) at a dose of 1000 mg/day for 1 week, and laser photobiomodulation using a diode laser with a wavelength of 635 nm (dose of 4 J per point, 2 points at each site) was performed one day before the procedure.

**Figure 1:**
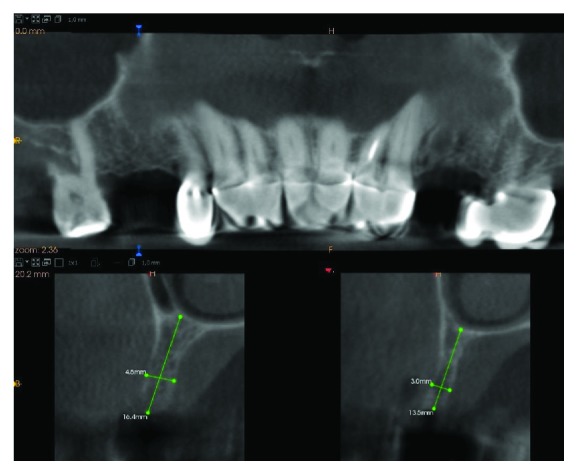
A CBCT scan of the patient with a very thin maxillary alveolar ridge.

Intraoral examination using CBCT (Kodak 9000 3D, Carestream/Trophy, Marne-la-Vallée, France) revealed that the volume of the ridge at the right side of the maxilla was 4.5 mm in width and 16.5 mm in height. At the opposite left side of the maxilla, the ridge amounted to 3.0 mm in diameter and 13.5 mm in height (Figure 1). A written informed consent form was signed by the patient before the treatment.

### 2.2. Surgical Phase and Prosthetic Rehabilitation

**Figure 2:**
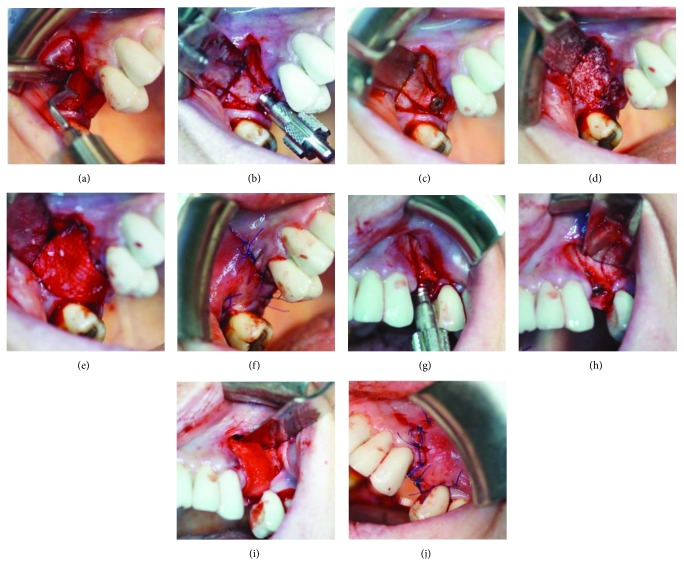
Ridge splitting with immediate placement of implants. (a) Bone osteotomy using piezosurgery. (b) Bone spreading with hand instruments. (c) The implant placement on the right side of the maxilla. (d) Alloplastic material grafting. (e) Collagen membrane placement. (f) Sutures. (g) Bone spreading with hand instruments on the left side of the maxilla. (h) The implant placement on the left side of the maxilla. (i) Placement of alloplastic material and collagen membrane. (j) Sutures.

The surgical procedure was conducted under local infiltrative anesthesia with articaine hydrochloride 4% plus epinephrine (Ubistesin®, 3M, USA). The access to the buccal and lingual part of the maxillary crests on both sides was prepared with a cold blade and a soft tissue elevator. Three cuts were conducted during the proceedings of the ridge splitting: one horizontal cut on the alveolar ridge and two vertical cuts on the buccal bone plate using the piezosurgery unit (Piezotome Solo, Acteon, New Jersey, USA) with a BS1 tip. In the first phase of the implant bed preparation, the Lindemann guide drill with a diameter of 2.2 mm was utilized; then, the ridge was split employing a bone spreader (Meisinger, Colorado, USA). In the last stage, the final drill with a diameter of 2.9 mm was used to prepare the implant bed, and two ICX-plus implants (ICX, Germany, USA) with a width of 3.45 mm and a length of 10 mm were placed. On both surgical sides, the guided bone regeneration was done using an alloplastic material (SinossGraft, Novadento, Italy) and a collagen membrane (SinossMem, Novadento, Italy) with a long disintegration time (5-6 months). The wound was protected using a nonabsorbable monofilament and an uncoated suture made of polyamide (Dafilon, B. Braun, Germany) with a size of 4.0 (Figure 2).

After the surgery, an antiseptic mouth rinse (chlorhexidine gluconate 0.12%, twice a day for 7 days) was directed and the patient was provided with the usual postsurgical indications (cold compresses in the first two days, antibiotic treatment with amoxicillin+clavulanic acid (Amoxiclav, Sandoz, Poland) at a dose of 1000 mg/day for 1 week, and nonsteroidal anti-inflammatory drugs, i.e., ibuprofen 200 mg, 3 times per day for 3 days).

**Figure 3:**
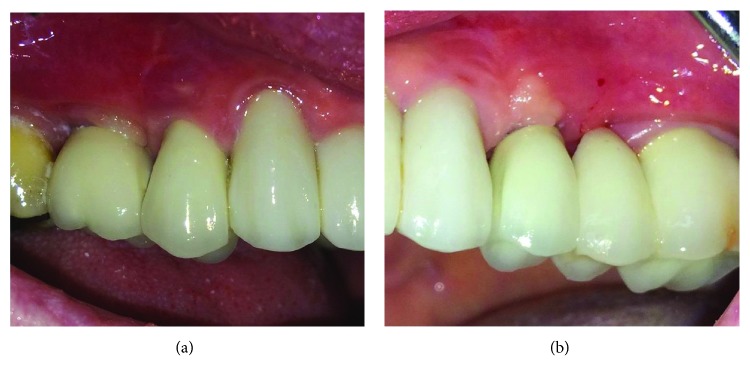
Prosthetic crowns immediately after cementation on the abutments. (a) Right side of the maxilla. (b) Left side of the maxilla.

Five months later, the implants were uncovered with use of a scalpel and healing screws were placed for two weeks. Next, the open tray method with prosthetic transfers was used to take the impression of the upper jaw. After ten days, the final porcelain crowns were made by a dental laboratory and cemented onto the implants (Figure 3).

### 2.3. Follow-Up Phase

**Figure 4:**
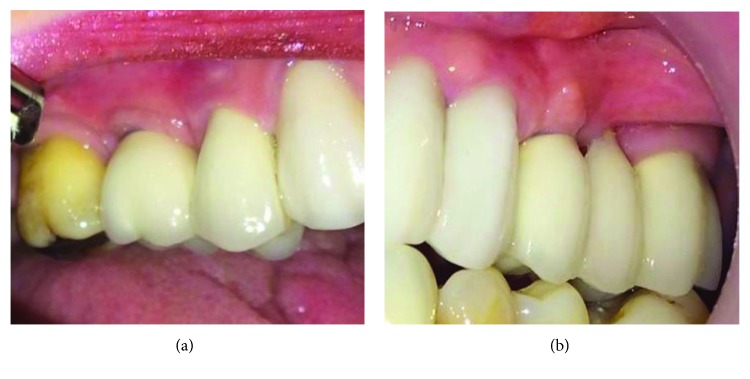
Prosthetic crowns 2 years after the cementation. (a) Right side of the maxilla. (b) Left side of the maxilla.

**Figure 5:**
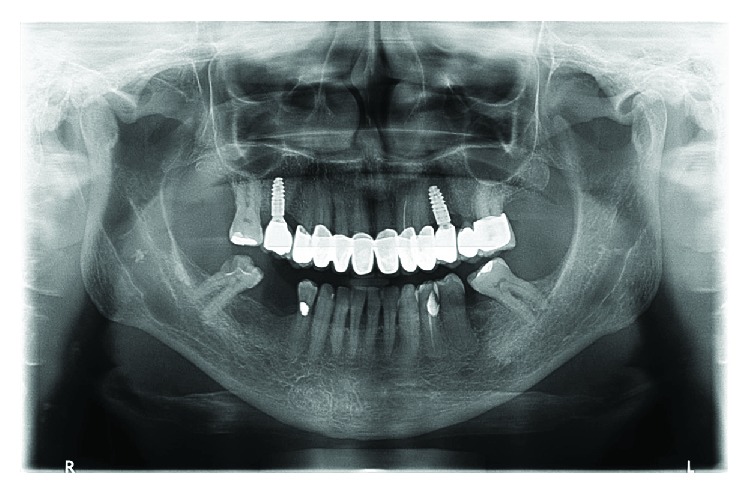
OPG 2 years after the treatment.

Every six months, the patient was referred for a check-up visit and the last control 24 months after the prosthetic crown placement showed a lack of bone loss in the collar part of both implants and the normal status of the peri-implant soft tissue without any signs of inflammation (Figures 4 and 5).

## 3. Discussion

Prevention of BRONJ/MRONJ in oral and maxillofacial surgery is critical in patients considered at risk. Proper oral hygiene, mouth rinsing with antibacterial fluids, and antibiotic and laser therapies are crucial factors to decrease the risk of bone necrosis after various surgical procedures in the oral cavities. According to the literature, the risk of BRONJ/MRONJ in patients taking oral bisphosphonates is low [16]. However, the risk of MRONJ for patients exposed to oral bisphosphonates in the management of osteoporosis after endodontic treatment, periodontal surgery, or implant placement is unknown [18]. Thus, the patients who need teeth restoration using dental implants and at the same time are treated with oral and especially intravenous bisphosphonates belong to a group with a higher risk of postsurgical complications.

In a recent systematic review, Khan et al. [16] showed that the incidence of BRONJ/MRONJ in the osteoporotic patient group ranged between 0.001% and 0.01%, similar to the percentage found in the general population (<0.001%). On the other hand, the occurrence of MRONJ/BRONJ is most significant in the oncology patient group that used high doses of bisphosphonates (1% to 15%). In the study conducted by Dodson in 2015 [18], the author concluded that MRONJ is an uncommon disease among patients exposed to antiresorptive or antiangiogenic medications. Furthermore, the risk of MRONJ among patients who are treated for osteoporosis using antiresorptive prescriptions is about 0.1% (range 0.004%–0.2%). The same risk for patients with cancer exposed to antiresorptive or antiangiogenic medicines is approximately 1% (range 0.2%–6.7%). Moreover, the risk of MRONJ for patients exposed to oral bisphosphonates in the management of osteoporosis after tooth extraction has amounted to 0.5% [18].

To know what bisphosphonate patients are taking along with the time duration is essential before any surgical approach in an oral cavity. Grant et al. [19] advised C-terminal telopeptide and collagen type 1 C test (CTx test) for patients receiving treatment for more than three years. The CTx test is a serum blood test allowing the observation of markers of bone turnover, and it may help in the examination and risk estimation to determine if MRONJ is developing.

Multiple methods are utilized for bone tissue augmentation [20]. The crest splitting technique allows placing an implant simultaneously with guided bone regeneration alternatively to horizontal and vertical augmentation procedures. It was proven that this method improves bone volume with a minimal failure rate [8]. The disadvantage of this procedure when using osteotomes for bone expansion is a fracturing of bone trabeculae that results in a decrease in peri-implant bone density and causes delayed secondary stability in contrast to the classical drilling technique. Trisi et al. [21] pointed out some bone necrosis on account of an obstruction of the Havers and Volkmann canals when using osteotomes. Furthermore, the temperature of bone increases by 10°C after using piezosurgery and the laser or surgical saw can lead to bone cell necrosis; thus, a proper cooling system of sufficient quality and experienced surgeons are crucial points if the surgery is to be performed safely [22, 23].

Admittedly, this paper is based on the therapy of an individual patient and does not involve the standardized measurement of a group. Within these limitations, the results imply that the method can be advantageous for patients taking bisphosphonates in the management of osteoporosis and it can be advantageous for an implantologist as well. Future randomized clinical trials considering a longer follow-up period are needed to support the safeness of this treatment.

## 4. Conclusion

The piezosurgery device has great potential to advance surgical techniques where accuracy in bone preparation is expected. Piezosurgery is useful and has safe tools for ridge splitting surgery on a very thin ridge (4.5 and 3.0 mm). Ridge splitting and guided bone regeneration with immediate implant placement can be safely performed on a patient taking bisphosphonates in the management of osteoporosis.
